# A balanced formula of essential amino acids promotes brain mitochondrial biogenesis and protects neurons from ischemic insult

**DOI:** 10.3389/fnins.2023.1197208

**Published:** 2023-06-15

**Authors:** Maurizio Ragni, Francesca Fenaroli, Chiara Ruocco, Agnese Segala, Giuseppe D’Antona, Enzo Nisoli, Alessandra Valerio

**Affiliations:** ^1^Center for Study and Research on Obesity, Department of Medical Biotechnology and Translational Medicine, University of Milan, Milan, Italy; ^2^Department of Molecular and Translational Medicine, Brescia University, Brescia, Italy; ^3^Department of Public Health, Experimental and Forensic Medicine, University of Pavia, Pavia, Italy

**Keywords:** mitochondrial biogenesis, aging, cerebral ischemia, essential amino acids, dietary supplementation, endothelial nitric oxide synthase, mammalian target of rapamycin

## Abstract

Mitochondrial dysfunction plays a key role in the aging process, and aging is a strong risk factor for neurodegenerative diseases or brain injury characterized by impairment of mitochondrial function. Among these, ischemic stroke is one of the leading causes of death and permanent disability worldwide. Pharmacological approaches for its prevention and therapy are limited. Although non-pharmacological interventions such as physical exercise, which promotes brain mitochondrial biogenesis, have been shown to exert preventive effects against ischemic stroke, regular feasibility is complex in older people, and nutraceutical strategies could be valuable alternatives. We show here that dietary supplementation with a balanced essential amino acid mixture (BCAAem) increased mitochondrial biogenesis and the endogenous antioxidant response in the hippocampus of middle-aged mice to an extent comparable to those elicited by treadmill exercise training, suggesting BCAAem as an effective exercise mimetic on brain mitochondrial health and disease prevention. *In vitro* BCAAem treatment directly exerted mitochondrial biogenic effects and induced antioxidant enzyme expression in primary mouse cortical neurons. Further, exposure to BCAAem protected cortical neurons from the ischemic damage induced by an *in vitro* model of cerebral ischemia (oxygen-glucose deprivation, OGD). BCAAem-mediated protection against OGD was abolished in the presence of rapamycin, Torin-1, or L-NAME, indicating the requirement of both mTOR and eNOS signaling pathways in the BCAAem effects. We propose BCAAem supplementation as an alternative to physical exercise to prevent brain mitochondrial derangements leading to neurodegeneration and as a nutraceutical intervention aiding recovery after cerebral ischemia in conjunction with conventional drugs.

## 1. Introduction

Aging is a natural process resulting from the progressive impairment of tissue and cellular activity, which leads to the loss of many biological functions. The brain is one of the most affected among the body’s organs. Neurodegenerative diseases are prevalent among older populations, heavily compromising their healthy lifespan (Azam et al., 2021). Major hallmarks of aging have been recently reviewed (López-Otín et al., 2023). In particular, brain mitochondrial function progressively deteriorates during aging due to multiple intertwined mechanisms (mtDNA mutations, defective proteostasis, reduced organelle turnover), which compromise mitochondrial bioenergetic function (i.e., ATP production) and enhance the production of reactive oxygen species (ROS) (López-Otín et al., 2023). Accumulation of ROS is indeed recognized as one of the most relevant causes of brain aging, and mitochondrial dysfunction plays a pivotal role in this process (Stefanatos and Sanz, 2018; Lima et al., 2022). The mitochondrial electron transport chain (ETC) is the primary cellular source of ROS, whereas mitochondrial DNA (mtDNA) is a primary target of oxidative attack. Neurons heavily rely on mitochondrial oxidative phosphorylation (OXPHOS) for energy production, making them highly sensitive to ROS production and oxidative stress (Nicholls and Budd, 2000). In rodent brains, the electron transfer rate at complex I and IV and mtDNA-encoded respiratory chain enzyme activities decrease with advancing age (Navarro and Boveris, 2007). In the human brain, transcriptional profiling revealed reduced expression of several genes involved in mitochondrial function, including the α subunit of the mitochondrial F1 ATP synthase (which couples oxidative phosphorylation to ATP synthesis) (Lu et al., 2004). Since ATP is required for DNA repair, this could contribute to mtDNA oxidative damage (Lu et al., 2004).

Besides being a known risk factor for several neurodegenerative diseases such as Alzheimer’s, Parkinson’s, and Huntington’s disease, which are all characterized by a reduction in mitochondrial function (Pantiya et al., 2020), aging is also the strongest and non-modifiable risk factor associated to ischemic stroke, which affects about 7.6 million of people worldwide every year (Feigin et al., 2021), with higher mortality and morbidity occurring in older than younger patients (Roy-O’Reilly and McCullough, 2018). During cerebral ischemia, insufficient cerebral blood flow, secondary to vessel occlusion, leads to a shortage of oxygen and glucose to the brain and severely impairs mitochondrial function. This results in ATP depletion, excessive ROS production, membrane depolarization, calcium overload, and excessive glutamate release with excitotoxicity (Liu et al., 2018; Chen et al., 2020).

Therefore, restoring mitochondrial function is crucial for neuron survival in ischemic conditions. Several approaches, both pharmacological (Valerio et al., 2011) or, more recently, based on mitochondrial transfer (Hayakawa et al., 2016; Chen et al., 2020; He et al., 2020) have been put forward and proved effective in protecting neurons from ischemic damage *in vitro* or *in vivo* mouse models. However, mitochondrial transfer has inherent technical challenges, and the pharmacological targeting of mitochondrial function in clinical trials has been unsuccessfully (Liu et al., 2018). Currently, the only treatment for ischemic attacks is limited to thrombolytic drugs like recombinant tissue plasminogen activator (rt-PA), which poses severe hemorrhagic risks and has a restricted therapeutic time window (Saver et al., 2013). Therefore, new therapeutic tools are required, and strategies to stimulate brain mitochondrial biogenesis could be valuable candidates (Valerio et al., 2011; Ranjan et al., 2020).

Healthy physical and dietary lifestyle habits’ influence in delaying aging is long known. A well-established anti-aging tool is physical exercise, which has beneficial outcomes on several age-related issues and is also effective in counteracting age-related cognitive impairment (Sofi et al., 2011). Observational studies found an inverse association between physical exercise and stroke risk in both men and women (Wannamethee and Shaper, 1992; Kiely et al., 1994; Ellekjær et al., 2000). A plethora of mechanisms could contribute to the beneficial effect of physical activity in preventing cerebral ischemia and ameliorating the outcome after ischemic stroke (Gubert and Hannan, 2021), and mitochondria play a central role.

Although the dynamics which mediate cell and organ adaptations to exercise are complex and multifactorial, mitochondrial remodeling is undoubtedly a central mechanism underlying its beneficial effects, not only in skeletal muscle or heart (Menshikova et al., 2006; Vettor et al., 2014) but also in the brain (Mattson, 2012; O’Reilly et al., 2023). Mitochondrial biogenesis is a complex phenomenon, mainly orchestrated by the transcriptional co-activator peroxisome proliferator-activated receptor-γ coactivator-1α (PGC-1α). PGC-1α interacts with and activates several transcription factors, including the Nuclear Respiratory Factors 1 and 2 (NRF1 and 2), that induce the transcription of genes of the OXPHOS pathway, and the mitochondrial transcription factor A (Tfam), which drives mtDNA transcription and replication, therefore activating mitochondrial biogenesis through the coordinated expression of both nuclear and mtDNA-encoded mitochondrial proteins (Scarpulla, 2008).

Stimulating exercise-induced mitochondrial biogenesis in the brain could represent a valuable strategy to combat aging-induced neurodegeneration (Mattson, 2012; O’Reilly et al., 2023). Among the signaling pathways recruited in the anti-aging and neuroprotective role of exercise-induced brain mitochondrial biogenesis, the mammalian target of rapamycin (mTOR), which has been shown to enhance synaptic plasticity, learning, and memory in response to exercise (Lloyd et al., 2017), is a good candidate. mTOR forms two complexes, mTOR complex 1 (mTORC1) and 2 (mTORC2), which display different protein composition and cellular targets. Several studies have demonstrated mTORC1-mediated transcriptional control of mitochondrial oxidative function through interactions with key regulators. mTORC1 controls PGC-1α function and its downstream targets and, in addition, can directly regulate mitochondrial function (Cunningham et al., 2007; Ramanathan and Schreiber, 2009). Furthermore, according to its role as a regulator of protein translation in response to amino acid abundance, mTORC1 stimulates mitochondrial biogenesis by enhancing the synthesis of nuclear-encoded mitochondrial proteins (Morita et al., 2013). Thus, mTORC1 promotes several aspects of brain mitochondrial metabolism, including oxygen consumption, ATP production, and mitochondrial biogenesis (Szwed et al., 2021).

Endothelial nitric oxide synthase (eNOS), by engaging cross-talk with both PGC-1α and mTOR, is another crucial regulator of mitochondrial biogenesis. eNOS produces the vasodilating agent nitric oxide (NO), thus matching the delivery of circulating nutrients/respiratory substrates with a corresponding increase in mitochondrial mass. Notably, the anti-aging effect of calorie restriction and the beneficial adaptations of the heart to exercise depend on the eNOS-mediated induction of mitochondrial biogenesis (Nisoli et al., 2005; Vettor et al., 2014).

Although the benefits of physical activity are well established, it is not always feasible for elderly persons, particularly in frail conditions such as after ischemic events. Therefore, an active research area investigates therapeutic targets and strategies for developing exercise mimetics, particularly in central nervous system disorders (Gubert and Hannan, 2021). We have shown that oral supplementation with a mixture of essential amino acids (EAAs) enriched with the branched-chain amino acids (BCAAs) leucine, isoleucine, and valine (BCAAem), activates mTOR and increases eNOS-mediated mitochondrial biogenesis and function in mouse heart and skeletal muscle, extending the average lifespan of middle-aged mice (D’Antona et al., 2010). Furthermore, feeding mice with an amino acid-defined diet enriched in EAAs, by activating mTOR in brown adipocytes induces mitochondrial thermogenesis and has a positive outcome on age-related diseases such as obesity and diabetes (Ruocco et al., 2020; Ragni et al., 2022). Most notably, BCAAem activates systemic mitochondrial bioenergetics and prevents both sarcopenia and decline in cognitive function in elderly malnourished patients (Buondonno et al., 2020).

On these bases, we have investigated whether BCAAem supplementation influences the mitochondrial biogenesis and antioxidant response in the hippocampus of middle-aged mice compared to those evoked by treadmill exercise training. Furthermore, we have also tested the protective effects of BCAAem applying oxygen-glucose deprivation (OGD) in murine primary cortical neurons. This is traditionally considered the most relevant *in vitro* model of ischemic stroke, causing neuronal injury sequelae consistent with the *in vivo* ischemia-reperfusion injury models. It allows investigating the pathways involved during ischemia and examining the resultant cellular damage (Holloway and Gavins, 2016; Trotman-Lucas and Gibson, 2021).

## 2. Materials and methods

### 2.1. Animals, diets, and treatments

*In vivo* studies were conducted as previously described (D’Antona et al., 2010) using middle-aged (16-month-old) male F2 Hybrid B6.129S2 (obtained from crossing C57BL/6J and 129S1/SvImJ) (Jackson Laboratory, Bar Harbor, ME, USA). Mice were treated according to the EU guidelines and with the approval of the Institutional Ethical Committee. Animals were given unrestricted access to a standard diet (4.3 kcal% fat, 18.8 kcal% protein, 76.9 kcal% carbohydrate, Laboratorio Dottori Piccioni, Gessate, Italy) and tap water. Amino acid content of the standard diet was (g/100 g mice food): arginine, 1.15; histidine, 0.48; isoleucine, 0.80; leucine, 1.50; lysine, 1.05; methionine + cysteine, 0.75; phenylalanine, 0.90; threonine, 0.75; tryptophan, 0.23; valine, 1.00. Methionine was supplemented to the basic mixture. Mice were divided into two groups, sedentary (*N* = 40) and exercise-trained (*N* = 40) groups, and each group was further subdivided into untreated and BCAAem-supplemented groups (20 mice/group). BCAAem supplementation (1.5 mg/g body weight/day in drinking water, with percent composition described in Table 1) was performed for 3 months. The amount of BCAAem to be dissolved in water was calculated by recording the average body weight and the average daily water consumption of each animal cage for 2 weeks before starting the treatment and regularly adjusted based on the same parameters. Body weight and drinking volume were checked weekly. Average food and water intake did not differ among the experimental groups. The exercise training was performed during the third month of supplementation, as previously described (D’Antona et al., 2010). Briefly, mice were trained on the belt of a 6-lane motorized treadmill (Exer 3/6 Treadmill, Columbus Instruments), supplied with shocker plates (electrical stimulus: 200 ms, 0.34 mA, 1 Hz). Mice first performed 2 days of a daily acclimation session of 20 min at 6 m/min; afterward, they ran 5 days/week with the following protocol: first week, 30 min at 10 m/min; second week, 60 min at 10 m/min; third and fourth week, 60 min at 12 m/min. The day after the last exercise training session, mice were sacrificed by cervical dislocation. Hippocampi were immediately dissected, frozen in liquid nitrogen, and stored at –80°C.

**TABLE 1:** Amino acid composition of the BCAAem mixture.

Amino acids	Percentage amount
L-Leucine	30.01
L-Lysine	19.58
L-Isoleucine	15
L-Valine	15
L-Threonine	8.4
L-Cysteine	3.6
L-Histidine	3.6
L-Phenylalanine	2.4
L-Methionine	1.2
L-Tyrosine	0.72
L-Tryptophan	0.48

Composition is expressed as a percentage of the total amino acid amount (g/100 g). BCAAem, branched-chain amino acid-enriched mixture.

### 2.2. Neuronal cultures and BCAAem treatment

Primary cortical neurons were prepared as previously described (Valerio et al., 2006) with minor modifications. Pregnant C57BL/6J mice were purchased from Charles River Laboratories (Calco, Italy). Fifteen-day mouse embryos were taken with caesarean section from anesthetized pregnant mice. Cortices were isolated, pooled, and mechanically dissociated into a single-cell suspension in Neurobasal medium (#21103-049, Invitrogen Corporation, Carlsbad, USA) containing 2% B27 supplement (#17504-044, Invitrogen), 500 μm glutamine (#ECB3000D Euroclone, Pero, Italy), 100 units/ml penicillin, and 100 μg/ml streptomycin (#ECB3001D, Euroclone) (N-B27 medium), and centrifuged for 5 min at 200 × *g*. Cells were plated onto poly-d-lysine (#P-0899 Sigma-Aldrich, Milan, Italy)-coated glass coverslips or dishes (2.5 or 10 × 10^4^ cells/cm^2,^ for confocal microscopy or all other studies, respectively) and cultured in N-B27 medium for 12 days as previously described (Valerio et al., 2011). Half of the N-B27 medium was changed 5 days after seeding. The BCAAem mixture was directly dissolved in the culture medium and added to the cells for mitochondrial biogenesis experiments after 9 days of *in vitro* differentiation. In preliminary time- and dose-dependence experiments, we found that 3 days of 0.5% BCAAem treatment exerted the maximal effect on mtDNA levels in primary cortical neurons (data not shown). Thus, the BCAAem mixture was used at the final concentration of 0.1% or 0.5% m/v, and cells were harvested and processed after 3 days of treatment.

### 2.3. Quantitative RT-PCR

For the analysis of mRNA levels (Valerio et al., 2011), total RNA was isolated using the RNeasy kit (Qiagen, Hilden, Germany). RNA concentration and purity were assessed with the NanoDrop 1000 (Thermo Scientific, Milan, Italy). 1 μg of total RNA was reverse transcribed using iScript cDNA Synthesis Kit (Bio-Rad Laboratories, Segrate, Italy). After the reverse transcription step, cDNA was diluted 1:5 in DNAse-free water, and 2 μL used for PCR amplification. Triplicate PCR reactions were performed on an iCycler iQ Real-Time PCR Detection System (Bio-Rad Laboratories). Relative gene expression was calculated by a comparative method (2^–ΔΔ*Ct*^) using GAPDH as a housekeeping gene. Primer sequences (Table 2) were designed using Beacon Designer 2.6 software (Premier Biosoft International, Palo Alto, CA, USA).

**TABLE 2:** Primers used for quantitative RT-PCR.

Gene	Forward primer	Reverse primer	PCR product (bp)	*T*_a_
*Pgc-1*α	ACTATGAATCAAGCCACTACAGAC	TTCATCCCTCTTGAGCCTTTCG	143	60
*Tfam*	AAGACCTCGTTCAGCATATAACATT	TTTTCCAAGCCTCATTTACAAGC	104	60
*CoxIV*	TGGGACTATGACAAGAATGAGTGG	TTAGCATGGACCATTGGATACGG	113	61
*CytC*	ATAGGGGCATGTCACCTCAAAC	GTGGTTAGCCATGACCTGAAAG	172	60
*eNOS*	AGCGGCTGGTACATGAGTTC	GATGAGGTTGTCCTGGTGTCC	116	60
*Sod1*	GGCTTCTCGTCTTGCTCTC	AACTGGTTCACCGCTTGC	153	60
*Sod2*	GCCTCCCAGACCTGCCTTAC	GTGGTACTTCTCCTCGGTGGCG	131	63
*Cat*	CACTGACGAGATGGCACACTTTG	TGGAGAACCGAACGGCAATAGG	173	63
*Gpx1*	TCTGGGACCTCGTGGACTG	CACTTCGCACTTCTCAAACAATG	157	62
*Gapdh*	TGACGTGCCGCCTGGAGAAA	AGTGTAGCCCAAGATGCCCTTCAG	98	63
*mtDNA*	ACATGCAAACCTCCATAGACCGG	TCACTGCTGAGTCCCGTGGG	131	62
*gDNA*	GGTCGCGGTGTGGGCATTTG	CGTGATCGTAGCGTCTGGTT	108	60

*Bp*, base pairs; *T*_a_, temperature of annealing; *Pgc*-*1*α, Peroxisome proliferator-activated receptor gamma coactivator 1-alpha; *Tfam*, Transcription Factor A, mitochondrial; *CoxIV*, cytochrome c oxidase subunit 411; *CytC*, cytochrome c; *eNOS*, endothelial nitric oxide synthase; *Sod1*, superoxide dismutase 1, cytosolic; *Sod2*, superoxide dismutase 2, mitochondrial; *Cat*, catalase; *Gpx1*; Glutathione Peroxidase 1; *Gapdh*, Glyceraldehyde 3-phosphate dehydrogenase. mtDNA, mitochondrial DNA. *gDNA*, genomic DNA.

### 2.4. mtDNA quantification

Mitochondrial DNA copy number was measured by quantitative PCR. Total DNA was extracted with a QIAamp DNA extraction kit (Qiagen), then amplified using primers specific for the mitochondrial D-Loop region and normalized to genomic DNA by amplifying the first intron of the β-actin nuclear gene. Primer sequences were designed using Beacon Designer 2.6 software and are listed in Table 2.

### 2.5. Citrate synthase activity

The enzyme activity was measured spectrophotometrically at 412 nm at 30°C in whole cell extracts (Valerio et al., 2011). Cell homogenates were added to a buffer containing 0.1 mM 5,5-dithio-bis-2-nitrobenzoic acid, 0.5 mM oxaloacetate, 50 mM EDTA, 0.31 mM acetyl CoA, 5 mM triethanolamine hydrochloride, and 0.1 M Tris–HCl, pH 8.1. Citrate synthase activity (measured as nmol citrate produced/min/mg protein) was expressed as fold change relative to the control set at 1.0.

### 2.6. Oxygen-glucose deprivation

Primary cortical neurons were transferred into glucose-free balanced salt solution (BSS) previously saturated with 95% N_2_/5% CO_2_ and incubated in an anaerobic chamber at 37°C for 3 h, as described (Valerio et al., 2011). Oxygen concentration was < 0.4% throughout the OGD period, as assessed by an oxygen analyzer (Servomex 580A, Crowborough, UK). Control neurons were transferred in BSS containing 5.5 mM glucose and incubated at 37°C under normoxic conditions (atmospheric oxygen, 5% CO_2_). The mTOR inhibitors rapamycin (#9904, Cell Signaling Technology, Leiden, The Netherlands) or Torin-1, (a gift of the Whitehead Institute for Biomedical Research, Cambridge, MA) and the eNOS inhibitor L-Nitro Arginine Methyl Ester (L-NAME) (#N5751, Sigma-Aldrich) were used at a final concentration of 100 nM, 150 nM, and 10 μM, respectively. Rapamycin, by binding the mTOR receptor FKBP1, inhibits mTORC1 access to substrates, while Torin-1 inhibits both mTORC1/2. All drugs were added 15 min before OGD and maintained during OGD and the subsequent 24-h recovery in culture medium in normoxia. The release of lactate dehydrogenase (LDH) in the culture medium was measured with the CytoTox 96Assay (G1780, Promega, Madison, WI, USA) as an index of neuronal death. To control for intra-experimental variations of cell number, LDH release values of each culture well were normalized to releasable LDH obtained by incubating the cells for 30 min with 1% Triton-X-100 at the end of each experiment. Duplicate LDH measurements were done on OGD experiments run in triplicate from at least three different cultures. OGD-mediated LDH release was compared to LDH release induced by 1 mM L-glutamate (Sigma-Aldrich) for 24 h and to maximal LDH release, as evaluated by 1% Triton-X 100 treatment of sister cultures.

### 2.7. Western blot

Frozen tissues were lysed in ice-cold RIPA buffer complemented with 1 × protease inhibitor cocktail (#11836153, Roche, Monza, MB, Italy). Protein concentrations were determined using the Pierce BCA protein assay reagent (#23227, Thermo Scientific). Lysates (30 μg) were separated on NuPage Bis-Tris 4-12% Pre-Cast gels (#NP0322BOX, Invitrogen) and transferred to nitrocellulose membranes (Whatman, Dassel, Germany). Primary antibodies rabbit anti-PGC-1α (1:1000, Chemicon #AB3242) and mouse anti-α-tubulin (1: 10,000, Sigma-Aldrich #T5168) were used with the appropriate secondary antibodies: respectively goat anti-rabbit IgG (H + L) IRDye 800CW (1:2500, #926-32211, LI-COR Biosciences, Bad Homburg, Germany); goat anti-mouse IgG (H + L) IRDye 680RD (1:3500, 926-68070, LI-COR Biosciences). Signals were acquired in dual infrared fluorescence with the Odyssey FC instrument (LI-COR Biosciences). Quantification was performed using Image Studio Lite software. The intensity of the bands was calculated from three immunoblots with samples from different cell preparations. Mean values were referred to as the control value, set at 1.0.

### 2.8. Confocal microscopy analysis of mitochondria

Cells were allowed to load MitoTracker Red CMXRos (#M7512) or MitoTracker Green (M7514) (both from Thermo Fisher Scientific) for 10 min, washed two times with Hank’s BSS containing calcium and magnesium, then fixed, counter-stained with Hoechst 33258 and mounted in Fluorsave (Calbiochem, Milan, Italy). MitoTracker Green was used to detect the total amount of mitochondria in cells, as it localizes to the organelle regardless of mitochondrial membrane potential. MitoTracker Red CMXRos was used to visualize only functional mitochondria, as its accumulation depends on membrane potential. Images were acquired using a ZEISS LSM 510 META confocal laser scanning microscope and analyzed by the LSM 5 software, version 3.5 (Carl Zeiss, Gottingen, Germany).

### 2.9. Statistical analysis

Data were analyzed by two-tailed unpaired Student’s *t*-test or one-way ANOVA with Bonferroni *post-hoc* test in instances of multiple comparisons. All data are reported as mean ± SEM unless otherwise stated. Statistical analyses were performed using GraphPad Prism version 4.0 software.

## 3. Results

### 3.1. BCAAem supplementation mimics the effects of physical exercise promoting mitochondrial biogenesis and antioxidant genes response in mice hippocampus

**FIGURE 1:**
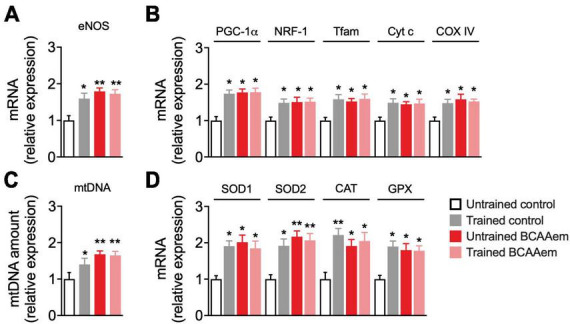
BCAAem increases the expression of mitochondrial biogenesis and anti-ROS genes *in vivo*. **(A)** RT-PCR of mRNA expression of endothelial nitric oxide synthase (eNOS) and **(B)** of the main mitochondrial biogenesis markers. **(C)** Mitochondrial DNA (mtDNA) amount. **(D)** mRNA expression of cytosolic and mitochondrial antioxidant genes. All data are presented as mean (*n* = 5) ± SEM. *p < 0.05 and **p < 0.01 vs. untrained control mice. Statistical analysis was performed with one-way ANOVA with *post-hoc* Bonferroni test.

We investigated whether dietary BCAAem supplementation could mimic the effect of physical exercise on mitochondrial biogenesis in the aged brain. To this end, we first analyzed mRNA expression of eNOS, a key regulator of mitochondrial biogenesis (Nisoli et al., 2003), which was necessary to mediate the BCAAem-induction of mitochondrial biogenesis in heart and skeletal muscles (D’Antona et al., 2010) and acted as an inducer of the protective hormetic response (Valerio and Nisoli, 2015). Dietary supplementation with BCAAem in untrained mice significantly increased eNOS expression to a similar extent to that induced by treadmill training (Figure 1A). Keeping with data from other groups (Steiner et al., 2011; Lezi et al., 2014), treadmill training promoted mitochondrial biogenesis in mice brain (Figures 1B, C). We found increased expression of the transcriptional coactivator PGC-1α, of the mitochondrial transcription factors NRF-1 and Tfam, and both nuclear- and mitochondrial-encoded subunits of the respiratory chain proteins cytochrome C (Cyt C) and cytochrome oxidase IV (COX IV) (Figure 1B) in mice hippocampi from trained mice, as compared to sedentary, untrained control. Further, mtDNA amount (an index of mitochondrial mass) was increased in hippocampi from trained mice (Figure 1C). As observed for eNOS, dietary supplementation with BCAAem in untrained mice was sufficient to significantly increase the expression of the mitochondrial transcription factors, respiratory chain proteins, and mtDNA to the same extent of those induced by exercise training in mice hippocampus (Figures 1B, C). Contrary to what we previously observed in heart and skeletal muscle, however, BCAAem supplementation did not further increase treadmill-training-induced mitochondrial biogenesis in mice hippocampi.

Protection from excessive ROS generation and oxidant damage is one of the primary mechanisms mediating the neuroprotective effect of physical exercise during aging (Lima et al., 2022). The expression of many ROS-detoxifying enzymes (including the mitochondrial SOD2 and those primarily present in the cytoplasm or peroxisomes such as GPX, SOD1, and catalase) is under the control of PGC-1α (St-Pierre et al., 2006). BCAAem supplementation significantly induced the expression of enzymes involved in the endogenous antioxidant response (SOD1, SOD2, catalase, and GPX) in hippocampi from sedentary mice. Remarkably, BCAAem supplementation-mediated induction of SOD1, SOD2, catalase, and GPX mRNA levels was comparable to that elicited by treadmill exercise training (Figure 1D). Again, BCAAem and treadmill exercise training in combination did not further increase the expression of all the antioxidant genes (Figure 1D).

### 3.2. Mitochondrial biogenesis and function are increased in mouse primary cortical neurons treated with BCAAem

**FIGURE 2:**
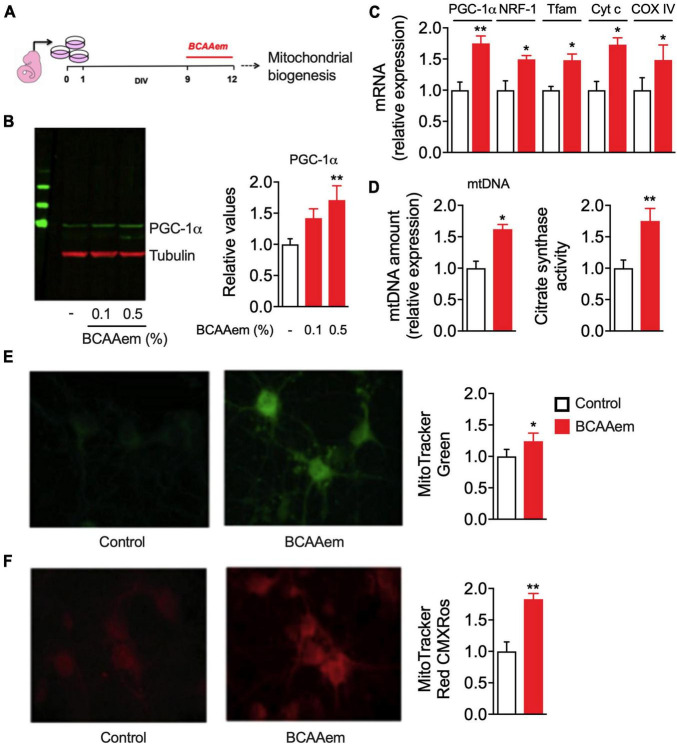
BCAAem directly activates mitochondrial activity in mouse primary cortical neurons. **(A)** Schematic picture of *in vitro* differentiation and treatment of cortical neurons. **(B)** Representative Western blot image and relative quantification of PGC-1α protein in untreated or BCAAem-treated cortical neurons at the indicated percentages. **(C)** RT-PCR of mitochondrial biogenesis markers in BCAAem-treated vs. control neurons. **(D)** Effect of BCAAem on mtDNA copy number and citrate synthase activity. **(E,F)** Representative confocal images of neurons loaded with mitotracker green **(E)** and mitotracker red **(F)** with relative quantification in the panels on the right. All analyses were performed on neurons (*n* = 4–5 independent cell plating) undergone the protocol described in panel **(A)**. All data are presented as mean ± SEM. Quantification of confocal samples with LSM 5 software was performed on at least five images. *p < 0.05 and **p < 0.01 vs. controls. Statistical analysis was performed with Student’s *t*-test **(D–F)** or one-way ANOVA with *post-hoc* Bonferroni test **(B,C)**.

Our data demonstrate that BCAAem supplementation increases mitochondrial biogenesis in mouse hippocampus *in vivo*. To verify if this EAA mixture can exert a cell-autonomous effect on cerebral neurons, we treated *in vitro* primary cortical neurons with BCAAem and analyzed mitochondrial biogenesis markers expression and activity. Primary cortical neurons were differentiated for 9 days. BCAAem was then added to the culture medium at the final concentrations of 0.1 or 0.5% (m/v) for 72 h, while the culture medium was only used in control samples (Figure 2A). As shown in Figure 2B, both BCAAem concentrations were effective in increasing PGC-1α protein expression in a dose-dependent manner; however, since only 0.5% reached statistical significance (*p* < 0.01 vs. control), this concentration was selected for subsequent experiments. As expected, and in line with the *in vivo* results, treatment with BCAAem 0.5% significantly increased the mRNA levels of the PGC-1α and its target gene NRF1 and those of Tfam, Cyt C, and CoxIV (Figure 2C). BCAAem treatment further increased mtDNA amount and citrate synthase activity (both indexes of mitochondrial mass) (Figure 2D). The latter finding was confirmed by the augmented fluorescent staining of mitochondria with MitoTracker Green (Figure 2E) in BCAAem-treated neurons. Most importantly, BCAAem also increased mitochondrial function, as indicated by the increased fluorescence of the Δψm-dependent dye MitoTracker Red MXRos (Figure 2F) in BCAAem-treated vs. untreated cortical neurons. Taken together, our results demonstrated that BCAAem, by acting through a direct and cell-autonomous mechanism of action, can activate neuronal mitochondrial biogenesis and activity.

### 3.3. BCAAem treatment protects cortical neurons from ischemic damage

**FIGURE 3:**
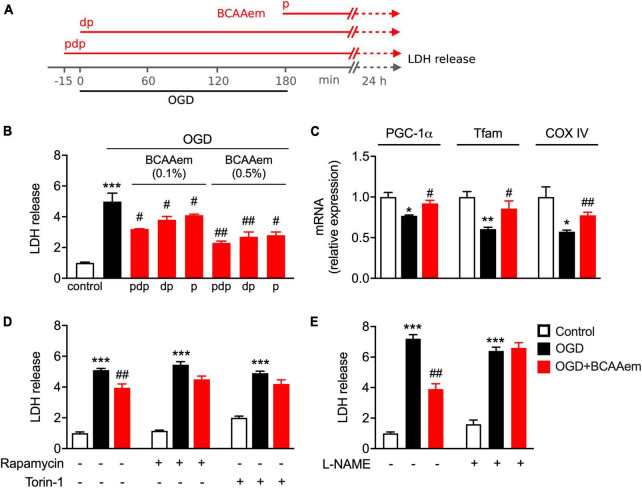
BCAAem treatment protects mouse cortical neurons from OGD through mTOR and eNOS and is associated with preserved mitochondrial biogenesis. **(A)** Schematic drawing of the timing applied for OGD and BCAAem treatment: pdp, pre, during, and post OGD; dp, during and post OGD; p, post OGD only. **(B)** Analysis of the ischemic damage (measured as LDH release) in cortical neurons in normoxic conditions (control) or subjected to OGD in the absence or presence of BCAAem doses for times as indicated in panel **(A)**. **(C)** RT-PCR of mitochondrial biogenesis markers measured after 24 h recovery in neurons in normoxia (white bars), subjected to OGD (black bars), or OGD plus BCAAem (0.5% pdp protocol, red bars). **(D,E)** LDH release in cortical neurons in normoxic condition (control), OGD condition, or OGD plus BCAAem (0.5% pdp protocol) with or without concomitant treatment with the indicated inhibitors. All data are presented as mean ± SEM from at least 4 independent neuronal preparations. *p < 0.05, **p < 0.01, ***p < 0.001 vs. control and ^#^*p* < 0.05, ^##^*p* < 0.01 vs. OGD. Statistical analysis was performed with one-way ANOVA with *post-hoc* Bonferroni test.

We previously demonstrated that pharmacological treatments preserving mitochondrial biogenesis protect primary cortical neurons from ischemic damage and reduce cerebral ischemic injury in mice subjected to permanent middle cerebral artery occlusion (Valerio et al., 2011). Therefore, we aimed to investigate if BCAAem–i.e., a potential exercise mimetic endowed with strong mitochondrial biogenic properties both *in vivo* and *in vitro*–could acutely exert a protective or therapeutic effect in a well-recognized model of neuronal ischemia (i.e., OGD in primary cortical neurons). Since the therapeutic intervention toward ischemic stroke has a well-defined temporal window (Saver et al., 2013), primary cortical neurons were supplemented with BCAAem in three different conditions: (i) starting 15 min before the OGD period, during the OGD exposure, and the 24 h of recovery after the OGD (pre-during-post, pdp), (ii) during OGD and the 24 h after OGD (during-post, dp), and (iii) only during the 24 h after OGD (post, p). The experimental procedure is summarized in Figure 3A. Following OGD, mouse cortical neurons undergo necrosis, whose extent is measured by the levels of cellular lactate dehydrogenase (LDH) released in the culture medium (Valerio et al., 2011). As expected, 24-h post-ischemic injury, the LDH amount released by oxygen-glucose-deprived OGD neurons was remarkably higher than controls (Figure 3B). Though not eliciting a complete reversal to the control (non-OGD) conditions, BCAAem treatment dose- and time-dependently reduced the necrosis induced by OGD, which was significantly blunted even at the lowest 0.1% concentration in the three different experimental conditions. While in our conditions, the highest level of protection was achieved in neurons that were preconditioned 15 min before the OGD with BCAAem 0.5% (pdp in Figure 3B), BCAAem exerted a significant protective effect against ischemic damage also when applied after the insult, suggesting its potential application as a therapeutic intervention. Notably, while OGD downregulated PGC-1α, Tfam, and COXIV expression, supplementation of BCAAem (0.5% in pdp condition) significantly restored their expression to almost control levels (Figure 3C), thus confirming that the BCAAem-induced protection from OGD was associated with induction of mitochondrial biogenesis.

### 3.4. Protection from OGD exerted by BCAAem requires both mTOR and eNOS

Next, we focused on the mechanism underlying BCAAem-mediated neuroprotection. We have previously shown that BCAAem supplementation extends the average life span and protects from age-related diseases in mice by boosting mitochondrial biogenesis and the endogenous antioxidant response acting through the mTOR/eNOS pathways (D’Antona et al., 2010; Ruocco et al., 2021). Since eNOS and mTOR signals are strictly intertwined (Pervin et al., 2007), we evaluated the involvement of both signaling pathways in BCAAem-mediated neuroprotection. Primary cortical neurons were pre-treated with rapamycin or Torin-1 (Thoreen et al., 2009), two selective mTOR inhibitors, or the eNOS inhibitor L-NAME before exposing cells to OGD in the absence or presence of BCAAem. As shown in Figure 3D, while supplementation with BCAAem significantly reduced the LDH levels induced by OGD, treatment with rapamycin significantly blunted the reduction in LDH release; moreover, Torin-1 was equally effective in blocking the BCAAem-induced decrease in LDH levels, thus confirming the involvement of mTOR complex 1 (mTORC1) in the mechanism of action of BCAAem-mediated neuroprotection. Notably, pre-treatment with L-NAME completely abolished the protective effect of BCAAem on OGD (Figure 3E). Our results, therefore, indicate that the protection from ischemic damage elicited by BCAAem requires both mTOR and eNOS signaling pathways.

## 4. Discussion

The benefit of physical exercise in delaying aging and age-related diseases is well-known (Garatachea et al., 2015). Notably, most - if not all - the disorders of aging are linked to impaired mitochondrial function (Bratic and Larsson, 2013; Cedikova et al., 2016; Son and Lee, 2019; Lima et al., 2022). Preservation of the age-related decline in mitochondrial mass and bioenergetic function, accompanied by a reduction in oxidative stress due to exercise-mediated hormetic effect, are key mechanisms of the anti-aging role of exercise (Menshikova et al., 2006; Nilsson and Tarnopolsky, 2019). Recognizing the healthy effects of exercise has prompted research toward the so-called exercise mimetics, reproducing the molecular mechanisms and signaling pathways of physical activity, primarily focusing on their beneficial impact on brain function (Gubert and Hannan, 2021). For more than a decade, we have been investigating a particular EAA formula (BCAAem), whose supplementation to sedentary middle-aged mice recovered mitochondrial biogenesis, induced the expression of antioxidant enzymes, and extended healthspan in an eNOS and mTOR-dependent manner (D’Antona et al., 2010; Ruocco et al., 2021). In a recent open-label randomized trial, we demonstrated that BCAAem also ameliorates the cognitive performance in aged subjects due to its capability to improve mitochondrial bioenergetics (Buondonno et al., 2020). We here show that BCAAem: (i) induces eNOS expression, mitochondrial biogenesis markers, and antioxidant genes in mouse hippocampus at levels comparable to those induced by exercise training; (ii) promotes mitochondrial biogenesis in mouse cortical neurons; and (iii) protects cortical neurons from an ischemic insult through mTOR- and eNOS-mediated mechanism(s).

Our *in vivo* data show that this particular EAA formula exerts exercise-mimetic effects on brain tissue without increasing the exercise-mediated effects. The reasons underlying this discrepancy with data from heart and skeletal muscle (D’Antona et al., 2010) are not clear. Still, they could be explained by the differences in mitochondria substrate utilization between muscle and brain during exercise. BCAAs are well-known to enhance skeletal muscle aerobic capacity by improving exercise-induced mitochondrial lipid oxidation (Gualano et al., 2011), possibly matched to a corresponding adaptive increase in mitochondrial biogenesis. The brain has, however, an intrinsic slow rate of β-oxidation (Schönfeld and Reiser, 2013), possibly explaining the lack of enhanced sensitivity to the mitochondrial biogenetic stimulus of BCAAem in the hippocampus in trained animals. Further experiments applying mice physical activity protocols less stressful than forced treadmill running (e.g., environmental enrichment and free-running wheels available throughout the period of dietary supplementation) (Love et al., 2022) could be more appropriate to investigate BCAAem’s capacity to enhance exercise benefits in advanced age.

Although the regulation of exercise-induced mitochondrial biogenesis has been extensively described in the skeletal muscle (Scarpulla, 2008), less is known about the signaling pathways operating in the brain; some hypotheses can be put forward. The release of lactate or myokines from muscle has been suggested to regulate exercise-induced mitochondrial biogenesis in the brain (Wrann et al., 2013; Park et al., 2021). However, our BCAAem mixture increased mitochondrial biogenesis and antioxidant gene expression in primary cortical neurons *in vitro*, supporting the hypothesis of a direct action of BCAAem in neurons. Further, BCAAem prevented the OGD-mediated impairment of mitochondrial biogenesis in cortical neurons. This occurs through activating the eNOS and mTOR pathways since protection from OGD-mediated damage was abolished in the presence of the mTOR inhibitors rapamycin and Torin-1 and by the eNOS- inhibitor L-NAME. The latter finding is particularly interesting given the well-known beneficial effects of eNOS-derived NO in counteracting cerebral ischemia (Endres et al., 2004). Notably, eNOS-dependent neovascularization has been implicated in physical activity-mediated improvement of long-term stroke outcomes (Gertz et al., 2006), strongly suggesting the role of exercise-mediated hormetic enhancement of the NO pathway in protection against brain injury (Faraci, 2006; Garry et al., 2015). Further, mTOR, eNOS, and PGC-1α are interconnected pathways modulating each other function (D’Antona et al., 2010; Craige et al., 2016), strengthening the proposal of BCAAem as a *bona fide* exercise mimetic. The high BCAA content of BCAAem could also support a direct role in both mitochondrial biogenesis and neuroprotective effects of the mixture; the brain has an oxidative capacity for BCAAs four-fold higher than that of muscles (Odessey and Goldberg, 1972) which suggests that their mitochondrial catabolism to acetyl-CoA in brain could directly activate the mitochondrial function. Moreover, BCAAem is enriched in EAAs whose catabolism generates mitochondrial Acetyl-CoA and/or can be used as anaplerotic mitochondrial substrates such as Lysine, Threonine, Histidine, Phenylalanine, Tyrosine, and Tryptophan.

During ischemic stroke, mitochondrial dysfunction resulting from the reduced oxygen supply impairs oxidative metabolism of glucose, with a switch to inefficient anaerobic glycolytic utilization; this metabolic failure then causes a drop in ATP production, an increase in ROS generation and, ultimately, cell death (He et al., 2020). Our data suggest that BCAAem supplementation, by providing neuron mitochondria with a readily oxidizable substrate, protects neurons from ischemic damage by efficiently recovering from the metabolic derangement induced by cerebral ischemia. Remarkably, dysregulation in nutrient sensing and increased metabolic inflexibility are major hallmarks of aging (López-Otín et al., 2023). Restoring the proper utilization of metabolic substrates through the improvement of mitochondrial function could therefore represent another of the mechanisms underlying the beneficial effects of BCAAem on age-related disease. Physical exercise is often an unaffordable option for elderly patients. The MATeR trial (Buondonno et al., 2020) demonstrated that, by sustaining mitochondrial bioenergetics, BCAAem prevents sarcopenia and cognitive decline in aged sedentary patients. The exercise-mimetic features of BCAAem could thus represent an alternative approach to prevent cerebral ischemia. Of note, patients often experience post-stroke cognitive and/or depressive symptoms. While the most frequent type of human cerebral ischemia involves the middle cerebral artery territory (spanning from the caudate and internal capsule to large cortical areas), during the post-stoke recovery phase, adaptive mechanisms occur, not only in the perilesional zone but also in more distal regions (Ceanga et al., 2021). One of these adaptive changes is hippocampal neurogenesis (i.e., proliferation, migration, and differentiation of neuronal progenitors). However, endogenous neurogenesis is a weak phenomenon (Gao et al., 2023), which can potentially be augmented by rehabilitation techniques. We recently demonstrated that complete neural stem cell differentiation is favored by a novel EAA-BCAA-based metabolic modulator activating mTORC1 and increasing mitochondrial function and ATP production (Bifari et al., 2020). The mTOR pathway positively affects bioenergetics and various aspects of neural plasticity involved in cognition and mood (Price et al., 2018). It has most recently proposed as a target to enhance endogenous neurogenesis after cerebral ischemia (Gao et al., 2023). Thus, we can speculate that the BCAAem-mediated mitochondrial biogenic effects, observed *in vivo* in mice hippocampus, could have a role in the “second therapeutic window” (Gao et al., 2023) for treating ischemic stroke and its long-term consequences.

A limitation of the present work is the absence of *in vivo* studies about the effects of BCAAem in ischemic animal models. We recently developed two novel EAA blends with slightly modified BCAA stoichiometric ratios, enriched with Krebs cycle intermediates, exerting greater efficacy than BCAAem on mitochondrial bioenergetics (Ruocco et al., 2020). We found a beneficial effect on physical and cognitive performance in progeroid SAMP8 mice supplemented with the PD-E07 formula, which promoted mitochondrial rejuvenation in muscles and the hippocampus (Brunetti et al., 2020). Further, the α5 formula, by inducing a persistent increase in energy metabolism, was required to promote the complete differentiation of neurons derived from murine neural stem cells or human induced pluripotent stem cells (Bifari et al., 2020). The latter formula also reduced neural tissue damage in a mouse model of severe contusive spinal cord injury (Dolci et al., 2022). Ischemic stroke is a medical emergency, and the pharmacological approach to reperfusion with intravenous rt-PA has a therapeutic window restricted within 4.5 h after stroke onset (Saver et al., 2013; Powers et al., 2018). Other strategies, including neuroprotective agents, stem cell-based therapies, or microbiota-gut-brain axis manipulation, are under investigation (Haupt et al., 2023). If confirmed with a future investigation using *in vivo* models of cerebral ischemia, EAA-BCAA-based metabolic modulators could be of interest in primary and secondary prevention or as a valuable aid in multidisciplinary post-stroke rehabilitation in humans.

## Data availability statement

The raw data supporting the conclusions of this article will be made available by the authors, without undue reservation.

## Ethics statement

The animal study was reviewed and approved by the Animal Welfare Committee (OPBA), University of Pavia.

## Author contributions

AV, GD’A, and EN conceived the study. GD’A and MR performed *in vivo* treatments. FF performed *in vitro* experiments and data analysis. MR wrote the original draft. CR and AS contributed to the data analysis and prepared the figures. AV, EN, AS, and MR revised the manuscript. All authors approved the submitted version.
